# Robotic-Assisted Transperitoneal Aortic Lymphadenectomy as Part of Staging Procedure for Gynaecological Malignancies: Single Institution Experience

**DOI:** 10.1155/2013/931318

**Published:** 2013-08-01

**Authors:** V. Zanagnolo, D. Rollo, T. Tomaselli, P. G. Rosenberg, L. Bocciolone, F. Landoni, G. Aletti, M. Peiretti, F. Sanguineti, A. Maggioni

**Affiliations:** ^1^Department of Gynecology, Cervical Cancer Center, European Institute of Oncology, Milan 20141, Italy; ^2^Department of Obstetrics and Gynaecology, Ospedale San Bortolo, Vicenza 36010, Italy; ^3^Department of Obstetrics and Gynaecology, Niguarda Ca' Granda Hospital, Milan 20162, Italy

## Abstract

*Introduction*. This study was designed to confirm the feasibility and safety of robotic-assisted transperitoneal aortic lymphadenectomy as part of staging procedure for gynecologic malignancies. *Methods*. Chart review of 51 patients who had undergone robotic staging with aortic lymphadenectomy for different gynaecologic malignancies was performed. *Results*. The primary diagnosis was as follows: 6 cases of endometrial cancer, 31 epithelial ovarian cancer, 9 nonepithelial ovarian cancer, 4 tubal cancer, and 1 cervical cancer. Median BMI was 23 kg/m^2^. Except for a single case of aortic lymphadenectomy only, both aortic and pelvic lymphadenectomies were performed at the time of the staging procedure. All the para-aortic lymphadenectomies were carried out to the level of the renal veinl but 6 cases were carried out to the level of the inferior mesenteric artery. Hysterectomy was performed in 24 patiens (47%). There was no conversion to LPT. The median console time was 285 (range 195–402) with a significant difference between patients who underwent hysterectomy and those who did not. The median estimated blood loss was 50 mL (range 20–200). The mean number of removed nodes was 29 ± 9.6. The mean number of pelvic nodes was 15 ± 7.6, whereas the mean number of para-aortic nodes was 14 ± 6.6. *Conclusions*. Robotic transperitoneal infrarenal aortic lymphadenectomy as part of staging procedure is feasible and can be safely performed. Additional trocars are needed when pelvic surgery is also performed.

## 1. Introduction

The feasibility and safety of robotically assisted para-aortic lymphadenectomy (PAL) have been already well reported, both with the robotic setup for pelvic surgery or with the sovrapubic approach [1, 2]. However, the upper limit, up to the left renal vein, is still debated, and technical aspects of PAL may differ depending on whether this procedure is the only one performed, or it is combined with other staging procedures for gynaecologic malignancies, such as pelvic lymphadenectomy, hysterectomy, omentectomy, and random peritoneal sampling.

The inframesenteric aortic nodes in most patients can be accessed and removed with the robotic setup for pelvic surgery. However, removal of the infrarenal aortic nodes up to the renal veins and, in particular, the left group can be very challenging. 

The infrarenal nodes have been reported as one of the most common site of nodal metastases in epithelial ovarian cancer, and recently they have been shown to be positive nodes in the absence of metastases in the ipsilateral inframesenteric nodes in endometrial cancer [3].

One of the major limitations of the current da Vinci robotic systems (Intuitive Surgical Inc., Sunnyvale, CA. USA) is its inability to provide access to the entire abdomen without relocating the robotic column. When removal of the infrarenal aortic nodes is required in case of full staging or excision, or both, of early or localized relapses of gynecologic malignancies, relocation of the robotic column may need to be performed. 

Magrina has shown, in his series of 33 patients, that robotic transperitoneal infrarenal aortic lymphadenectomy can be performed adequately and safely with the robotic column at the patient's head. Operating table/robotic column rotation and additional trocar sites are needed when used in conjunction with robotic pelvic surgery [2].

## 2. Material and Methods

The technique described by Magrina of robotic transperitoneal infrarenal aortic lymphadenectomy, with few minor modifications, was performed on 51 patients who underwent surgical treatment between January 2007 and October 2012 for epithelial ovarian (*n* = 31), endometrial (*n* = 6), cervical (*n* = 1), tubal (*n* = 4), and nonepithelial ovarian cancers (*n* = 9); among the last cases, 7 were dysgerminomas, 1 immature theratoma, and 1 neuroectodermic tumor (Table 1).

Except for a single patient who underwent aortic lymphadenectomy only, both aortic and pelvic lymphadenectomies were performed at the time of the staging procedure. All of para-aortic lymphadenectomies were carried out to the level of the renal veins but in 6 cases where the dissection was performed to the inferior mesenteric artery. Hysterectomy was performed in 24 patients (47%). Most of the times infracolic omentectomy was performed laparoscopically prior to robotic docking.

Intraoperative data were prospectively recorded. Perioperative data were extracted from electronic patient records and included patient age and body mass index (BMI), total operating time, (total console time, aortic lymphadenectomy console time, docking time, and table rotation time), number of aortic lymph nodes removed, additional procedures, and intraoperative and postoperative complications. The IEO Institutional Review Board approved the study.

Rotation time was defined as the time to rotate the operating table, from completion of pelvic surgery and un docking to completion of table rotation, for the last 20 cases we rotate the robotic column instead of the operating table. Docking time was defined as the time to advance the robotic column and attach the robotic arms to the trocars for the aortic lymphadenectomy. Console time was defined as the time when the surgeon sat at the robotic console for performance of the aortic lymphadenectomy.

All statistical analyses were performed using Microsoft Excel 2007. To estimate continuous variables, Student's *t*-test was used. All *P*-values presented are two-sided, and associations are considered significant if the *P*-value is <0.05. 

### 2.1. Surgical Technique

The surgical technique is the one described by Magrina et al. [2] with a few small modifications that we have added during our learning curve. We used both the S and Si Da Vinci System depending on their availability. 

**Figure 1:**
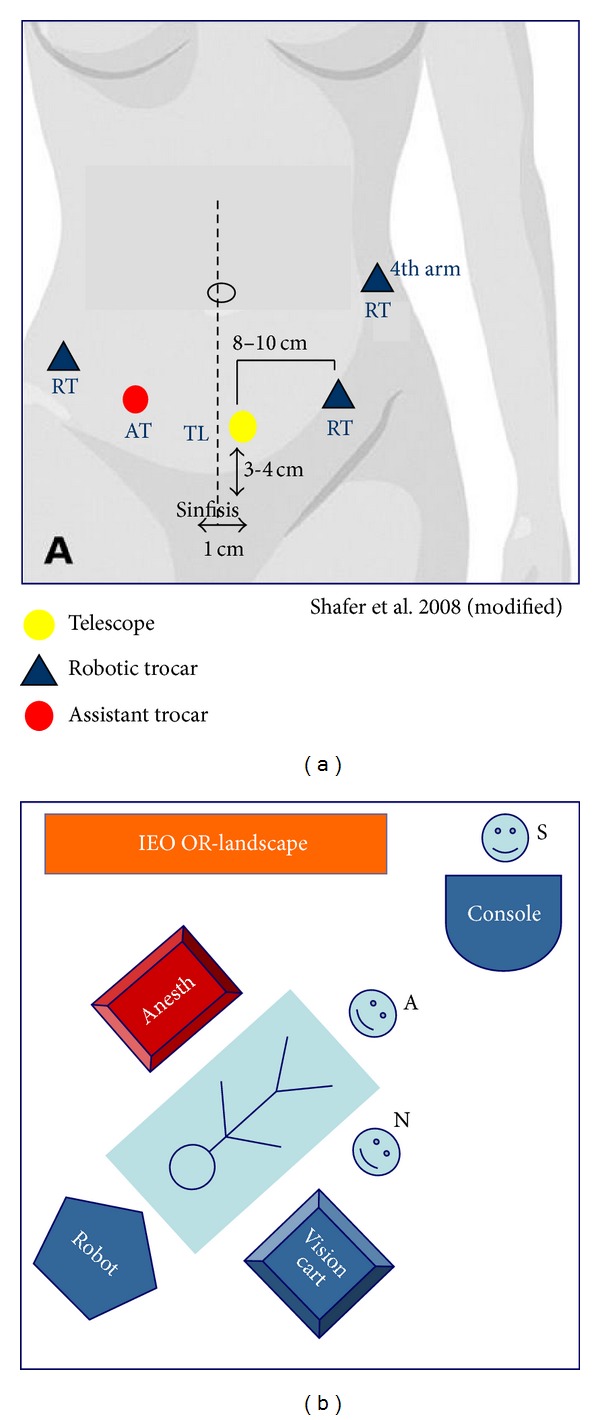
Trocar sites and OR landscape for paraortic lymphadenectomy [4].

The trocar placement for the pelvic portion of the robotic operation has been previously described. A new set of trocars was placed in the lower pelvis for the infrarenal aortic lymphadenectomy after pelvic surgery (Figure 1). An optical 12 mm trocar was inserted 3 or 4 cm suprapubically and 1 or 2 cm to the left of the midline. Two robotic trocars were inserted 10 to 12 cm to the right and the left of the optical trocar, a third robotic trocar is placed 10 to 12 cm the left of the umbilicus. Two of the robotic arms are used for the pelvic approach as well to therefore reducing the total number of trocar sites. A monopolar scissors (EndoWrist Hot Shears; Intuitive Surgical, Inc.; surgeon dependent) was used on the right robotic arm or on the left one in case of a left-handed surgeon, and a bipolar grasper (EndoWrist Maryland Forceps; Intuitive Surgical, Inc.) was used on the left robotic arm or vice versa for a left-handed surgeon. For the first few cases two accessory trocars were placed 2 cm caudally and equidistant to the right and left of the optical trocar, afterwards only one accessory trocar was placed equidistant between the sovrapubic optical trocar and the left robotic trocar.

The patient was placed in Trendelenburg position, and the robotic column was positioned at the patient's head. The assistant stood between the patient's legs and when we used 2 accessory trocars he/she used the left hand to retract the duodenum and pancreas ventrally with a 10 mm fan bowel retractor (Autosuture Endo Retract II; Tyco Healthcare Group LP, Norwalk, CT, USA) introduced through the left assistant trocar and the right hand for lateral retraction of the sigmoid mesentery, insertion of a vessel-sealing and cutting device, and suction and irrigation using the right assistant trocar. Since we have started to use the 4th arm with a fenestrated grasper (EndoWrist Cadier Grasper; Intuitive Surgical, Inc) to retract the duodenum ventrally, the 2nd assistant trocar to the right of the optical trocar is not placed anymore and the assistant uses the accessory port to the left.

A small (3-4 cm) incision was made on the peritoneum overlying the midportion of the right common iliac artery and extended to the aortic bifurcation. A small tent was then created by gently elevating the peritoneum ventrally with the 4th arm gasping forceps, preventing the small bowel from sliding into the surgical field.

After identifying the right ureter the right aortic nodes over the vena cava were excised first, as well as the interaortic nodes. The dissection was extended cranially until no nodal tissue was present, usually at or above the level of the insertion of the right ovarian vein to the vena cava.

To access the inframesenteric left aortic nodes, the surgeon extended the peritoneal incision from the aortic bifurcation caudally and over the left common iliac artery for approximately 4 to 5 cm.

The sigmoid mesentery was retracted laterally by the assistant surgeon, exposing the psoas muscle and the left ureter. The left inframesenteric nodes were then removed.

The inferior mesenteric artery, when necessary, was transected with a tissue-sealing device to increase exposure by allowing additional lateral mobilization of the left colon mesentery and facilitating the removal of the left infrarenal nodes. The left ovarian vein and the cranial border of the left renal vein were the lateral and upper limits of left aortic dissection, respectively.

## 3. Results

The patients' mean age was 41 years (range 18–59), and the mean BMI was 23.0 kg/m^2^ (range 18–33 kg/m^2^). The mean time for table rotation was less than 15 minutes. Most of the time was employed for preparation; table rotation itself lasted less than 60 seconds, during which time the ventilatory support was discontinued without major changes in patient's oxygen saturation. While robotic column rotation lasted less than 5 minutes, the mean docking time was 5.0 minutes (range, 2 and 15 minutes).

Median total time for procedures was 285 minutes (range 192–403 minutes), the median console time was 250 minutes (range 142–350 minutes) with a significance difference (*P* = 0.02) between patients who underwent hysterectomy (301 minutes) and those who did not (270 minutes). For aortic lymphadenectomy the median console time was 110 minutes (range 64.5–180 minutes) (Table 2).

The mean blood loss was 50 mL (range, 20–200 mL).

In 8 cases the procedure lasted more than 360 minutes: 1 patient with endometrial cancer involving the cervix who underwent radical hysterectomy, in the other 7 cases the extra operative time was due to extensive lyses of adhesions or intraoperative complications.

Excluding those 8 cases displaying a significant longer operative time: median total time for procedures was 277 minutes (range 212–330 min.), and the median console time was 240 minutes (range 131–290 minutes).

There were 17 nonsystematic lymphadenectomies: 11 monolateral para-aortic lymphadenectomies (either right aortic nodes over the vena cava plus interaortic nodes or interaortic plus inframesenteric/infrarenal nodes), and 6 inframesenteric lymphadenectomies, the remaining 34 were complete systematic lymphadenectomies. The mean number of nodes was 29.2 ± 9.6 with median number of pelvic nodes of 15 ± 7.6, whereas the mean number of para-aortic nodes was 14 ± 6.6. In the group of systematic lymphadenectomies the mean number of pelvic lymph nodes is 20 ± 5.5 and of para-aortic is 15 ± 5.5 (Table 3). The mean number of positive nodes was 0.37 ± 1.13. 

The mean number of aortic nodes collected with systematic pelvic and aortic lymphadenectomy comparing our first 10 and final 10 patients was 15 (±4.9) in first group and 16 (±5.5) in second group (*P* = 0.5).

In 8 patients with a BMI ≥28 kg/m^2^ the mean number of aortic nodes was 13 ± 7.4. Due to the small number of cases we did not compare this group of patients with those with BMI <25 kg/m^2^.

Table 4 summarizes intraoperative and post-operative complications: 2 patients had significant intraoperative bleeding (>500 mL) one from the vena cava and the other one from a lumbar artery, both controlled robotically. We did not have any conversion to laparotomy. Seven cases of chylous ascites were observed in the immediate post-operative course: 4 of the cases improved with low-fat diet only, on the contrary the other 3 patients required total parenteral nutrition for a few days while fasting.

The mean length of hospital stay for all surgical procedures was 3.0 days (range 2–7.5 days). 

Late postoperative complications that could be related to pelvic and aortic lymphadenectomy included pelvic and aortic lymph cyst formation in 3 patients, which resolved conservatively. Other postoperative complications were: one case of ureteral fistula which was treated by ureteral stent placement, two cases of port-site hernia, of which one required a reintervention, and mild to moderate legs edema in three cases.

Median followup was 26 months (range 1–56 months). During followup the six cases of recurrence were observed: one port-site recurrence in a patient with epithelial ovarian cancer FIGO stage IC after 30 months, this patient had a second recurrence (carcinomatosis) after 22 months; a second patient with ovarian cancer FIGO stage IIB had a recurrence (carcinomatosis) after 6 months, and she died of the disease after 8 months. A third patient with ovarian cancer FIGO stage IB had a spleen recurrence, treated by splenectomy. A fourth patient with ovarian cancer FIGO stage IIC had a liver recurrence after 33 months, and at then a patient with IA high grade ovarian cancer presented with lymphnode recurrence. 

## 4. Discussion

Gynecology oncologists still have some disagreements concerning Para-Aortic Lymphadenectomy (PAL) for gynecologic malignancies such as its therapeutic role, the upper limit of dissection: inferior mesenteric artery versus left renal vein, and indications of different surgical approaches: traditional versus minimally invasive one.

The inframesenteric aortic nodes in most patients can be accessed and removed with the robotic setup for pelvic surgery, by placing the trocars higher than usual and leaving the robot column between the patient's legs at all times [14]. However, removal of the infrarenal aortic nodes up to the renal vein and, in particular, the left group is difficult, can be incomplete, or can be unsafe due to the steep orientation of the robotic instruments in such a setting and the proximity between the optic and the renal vein (just beneath the camera port site). 

Therefore, exposure of the upper limit (left renal vein in our practice) as described by Lambaudie et al. [1] was sometimes difficult, particularly in case of high BMI. This difficulty in exposing the higher part of the dissection (between the left renal vein and inferior mesenteric artery) could explain the lower number of lymph nodes observed in this group compared with isolated PAL (7.8 versus 14.6) where the sovrapubic approach was chosen.

There is no doubt that the left infrarenal nodes are the most difficult to remove, both in laparoscopy and with the robotic approach, and are located in an area with potential vessel anomalies and therefore at higher risk of vascular injuries. 

Khöler et al. [15] evaluated the feasibility and oncologic value of laparoscopic transperitoneal pelvic and para-aortic lymphadenectomy in 650 patients; to confirm the complexity of the laparoscopic procedure the yield of a mean of 15 lymph nodes in para-aortic lymphadenectomy was associated with a learning curve of more than 100 procedures and right-sided para-aortic, left-sided inframesenteric and left-sided infrarenal lymphadenectomy took an average of 36, 28, and 62 min, respectively. The procedure can be even more challenging in patients with previous abdominal surgery and short small-bowel mesentery or in obese patients.

This is, to our knowledge, the largest published series of robotic-assisted transperitoneal aortic lymphadenectomy using the sovrapubic approach as described by Magrina et al. [2].

Magrina's trial of aortic lymphadenectomy with 2 female cadavers showed that to safely and expeditiously remove the infrarenal aortic nodes up to the renal vessels and, in particular, the left group, it was necessary to place the robotic column at the patient's head and the trocars in the lower pelvis. In his series of 33 patients the mean number of nodes was 12.9 (range, 2–27); the mean number of positive nodes was 2.6 (range 0–8) and there was one conversion to laparotomy.

Similarly in our series of 51 patients the mean number of nodes was 14 ± 6.6. In the subgroup that underwent a complete systematic lymphadenectomies the mean number of pelvic lymphnodes was 20 ± 5.5 and of para-aortic was 15 ± 5.5. The mean number of positive nodes was 0.37 ± 1.13, and there were no conversions to laparotomy. Our data compare favourably with the data available in the literature (Table 5).

Division of the inferior mesenteric artery, performed in our series only when needed, can markedly improve access to and exposure of the left infrarenal nodes without resulting in any kind of complications as confirmed by colorectal surgeons practice when they want to obtain a tension-free colorectal anastomosis in the presence of a short inferior mesenteric artery.

At the beginning of our experience, following Magrina's technique, we performed the rotation of the operating table, that, even though was devoid of complications, was always cumbersome and stressful for the anesthesiologists and the operating room personnel. At the present time our operating room nurses are very well trained in the rotation of the robotic column that it seems to be less stressful and takes a similar time as the rotation of the operating table. With the robotic column at the patient's head, we observed that additional upper-abdominal procedures such as omentectomy, diaphragmatic biopsies or appendectomy could also be performed through the same robotic trocar placement.

Our console times (mean time of 110 minutes) for infrarenal transperitoneal lymphadenectomy are higher compared to those published by Magrina et al. (mean console time of 42 minutes) but similar to the ones published by other authors. Fastrez et al. [16] observed a median operation time for para-aortic lymphadenectomy (PAL) of 137.5 min (90–185 min). Lambaudie et al. [1] in his series of 39 cases of isolated transperitoneal PAL described an operative time of 166.5 and a lymphnode yield of 13.3.

Although a comparison with laparoscopic times is not correct because of different logistics and setups of robotics, a mean of laparoscopic times of 60 and 64 minutes for the inframesenteric technique and 98 minutes for the infrarenal approach was published [15, 17, 18].

Our mean number of aortic nodes is 14.0 and it compares favourably with the mean of 10.9 of the laparoscopic approach as discussed in Magrina's paper [19–22] and with the figures published by different authors with the robotic setup for pelvic surgery (Figure 1).

The mean number of aortic nodes in systematic lymphadenectomies between our first 10 and final 10 patients is 15 versus 16 respectively, this observation confirms one of the most important advantages of robotic technology compared to the traditional laparoscopic approach such as a much shorter learning curve, moreover for surgeons that may have a very little or none previous laparoscopic expertise. Köhler [15] in fact described with the laparoscopic approach, an increase in the number of aortic nodes, from 5.5 to 18.5, during a period of 9 years and the establishment of a surgical protocol for laparoscopic aortic lymphadenectomies, as mentioned earlier, required 100 procedures in his expert hands.

The occurrence of major vessel injuries in our study (4.8%) was similar to incidence (6%) described by Magrina in his series [2] and the one (4.6%) reported by Possover et al. [17] with inframesenteric aortic lymphadenectomy. Control of major vessel bleeding is facilitated by the robotic instrumentation when the bleeding site can be reached, in fact robotic grasper controlling the bleeding can be left in place while preparations for haemostasis are being made.

The main advantages of the robotic technology continue to be the increased precision, accuracy, and articulation of the robotic instruments and the sitting position of the surgeon, with a stereoscopic image [23] facilitating minimally invasive surgery.

In terms of comparison of robotic-assisted procedure to the open approach and the laparoscopic one we reviewed the literature reported complications for the different surgical approaches (Tables 6 and 7).

Multiple reports showed (Table 6), beside a significantly positive impact on the perioperative outcomes, a significant drop in complication rate when robotic-assisted surgical management of endometrial cancer patients (including pelvic and para-aortic lymphadenectomy as well) was compared to the traditional laparotomic approach. A similar trend (Table 7) was observed when the robotic approach was compared to laparoscopy for the treatment of the same type of patients.

The present robotic system using the sovrapubic approach (Magrina's technique) has inherent disadvantages for infrarenal aortic lymphadenectomy, when combined with other staging procedures, in particular the operating table or the robotic column must be rotated of 180 degrees and additional trocars must be inserted, however these extra steps and extra operative time do not seem to delay the patient's recovery. It should be underlined that the incision required for the traditional LPT approach would be xifo-pubic and therefore having a few more sovrapubic port sites does not seem to jeopardize the positive impact on patients quality of life of the robotic-assisted procedure compared to the open surgery, especially for higher BMI patients. Table/robotic column rotation requires coordination between the operating team and the perioperative and anaesthesia personnel but such a coordination allows to significantly reduce the time needed to accomplished those extra manoeuvres. 

Beside the longer consol time the addition of an aortic lymphadenectomy to pelvic surgery did not increase robotic costs because the additional robotic and the assistant trocars are reusable and the robotic instruments are the same ones already used for pelvic procedures.

In our series most of the subjects had a BMI <25 kg/m^2^ and therefore we did not face the challenge that obesity placed to the performance of an aortic lymphadenectomy, such a challenge is well experienced for the traditional laparoscopic approach. Obesity is described, in fact, as the most common reason for conversion with the laparoscopic approach, particularly in patients with a BMI higher than 35 kg/m^2^ [24].

In Magrina's series [2] robotic approach in patients with BMI >25 kg/m^2^ resulted in a lower number of aortic nodes than in patients who had a normal BMI (12.8 versus 5.2 resp.), usually because of unsatisfactory exposure. Other authors did not confirm this trend even though the operating time seems to be longer in the obese groups [24–27]. Similarly in our series in the overweight group (BMI ≥ 28 kg/m^2^), even though small, there were no conversions to LPT and the mean number of aortic nodes was not significantly smaller than the number yielded in patients with BMI <28 kg/m^2^, confirming the positive impact of the robotic approach in obese subjects who are the ones that may benefit the most from minimally invasive surgery. 

In conclusion, our data confirm that robotic infrarenal aortic lymphadenectomy performed with the sovrapubic approach is feasible, safe, and oncologically adequate when performed with the robotic column at the patient's head. The operating time was acceptable and comparable with previously published data, and the number of aortic nodes was similar or slightly better for sovra-pubic robotic surgery approach compared to robotic setup for pelvic surgery and laparoscopy. The major limitation of the study is due to the retrospective nature of the data collection that, as it is well known, may hold a very high bias for under reporting complications especially minor complications, however the data available in the literature were of the same nature. 

Major disadvantages to this approach are the need for operative table/robotic column rotation and additional trocar sites placement when the infrarenal aortic lymphadenectomy is performed as a part of staging procedures. Future robotic technology should facilitate operations in all 4 abdominal quadrants therefore avoiding table/robotic column rotation, additional trocars placement, shortening operative time and farther improving patients' quality of life.

## Figures and Tables

**Table 1:** Patients characteristics and hysthology.

Population characteristics	Numbers
Age	41 (range 18–59)
BMI	23 (range 18–33)
Tumor:	
Endometrial cancer	6
Tubal cancer	4
Cervical cancer	1
Epithelial ovarian cancer:	31
Clear cell	9
Endometrioid	8
Serous	8
Squamous	2
Mixed	1
Mucinous	1
Indifferentiated	1
NA	1
Nonepithelial ovarian cancer	9
Dysgerminoma	7
Immature teratoma	1
Neuroendocrine tumor	1

**Table 2:** Operation and console time.

Procedure	Total operative time (min)	*P* value	Console time (min)	*P* value
Pelvic + LA plus hysterectomy	301	0.02	270	0.12
Pelvic + LA without hysterectomy	270		240	

**Table 3:** Mean number of lymph nodes in different groups.

	Pelvic lymph nodes mean (sd)	LA lymph nodes mean (sd)
Total lymphadenectomies (Pts = 51)	15 (±7)	14 (±6)
Systematic lymphadenectomies (Pts = 34)	20 (±5)	15 (±5.5)
Nonsystematic lymphadenectomies (Pts = 31)	11.3 (±4) (Pts = 28)	12 (±6) (Pts = 14)
Obese pts (Pts = 8)	17 (±10)	13 (±7)

**Table 4:** Intraoperative and postoperative complications.

Complications	No. pts (%)
Intraoperative complications	
Significant Bleeding (>500 mL)	2 (3.9)
Conversion rate	0
Postoperative complications	
Trasfusion rate	3 (5.8)
Chylous ascites	7 (13.7)
Vaginal leakage	2 (3.9)
Ureteral fistula	1 (1.9)
Femoral nerve injury	1 (1.9)
Legs edema G1-G2	4 (7.8)
Port-site hernia	2 (3.9)
Lymphocele	4 (7.8)
Lymphatic ascites	1 (1.9)
Total	**25 **

**Table 5:** Literature review.

Author	OR Time (min)	EBL (mL)	LOS (day)	Para-aortic nodes (*n*)	Conversion (%)	Complication (%)
Boggess et al. (2008)*N* = 377 [5]	283	47	1.4	6	2.9	6.4
Magrina et al. (2011) *N* = 67 [6]	182	141	1.9	8.7	2.9	12.0
Holloway and Ahmad (2012) *N* = 65 [7]	186	115	1.25	7.7	NA	1.54
DeNardis et al. (2008) *N* = 56 [8]	177	105	1.0	6.5	5.3	14.2
Lambaudie et al. (2012) *N* = 39 [1]	160	112	2.9	14.6	2.2	5.0
Backes et al. (2012) *N* = 315 [9]	/	100	1	8.8	6.3	7.2

**Table 6:** Robotic versus laparotomic hysterectomy and staging endometrial cancer.

Robotic versus Laparotomic	Boggess et al. (2008) [5] *N* = 105 versus 138	Elsahwi et al. (2012) [10] *N* = 155 versus 150	Magrina et al. (2011) [6] *N* = 67 versus 99	Paley et al. (2011) [11] *N* = 377 versus 131
BMI (Kg/m^2^)	33 versus 35	34.5 versus 33	30.7 versus 30.5	NA
OP time (min)	191 versus 147*	127 versus 141*	182 versus 163	283 versus 139*
EBL (mL)	75 versus 266*	119 versus 155*	141 versus 472*	47 versus 198*
LOS (day)	1.0 versus 4.4*	1.5 versus 4*	1.9 versus 5.6*	1.4 versus 5.3*
Nodes (*n*)	33 versus 15*	20 versus 20	25 versus 31*	16 versus 13
Complication (%)	5.8 versus 29.7*	10 versus 27*	12 versus 30*	6.4 versus 20.6*

*P < .001 (mean values).

**Table 7:** Robotic versus laparoscopic hysterectomy and staging: endometrial cancer.

Robotic versus LPS	Bogges et al. (2008) [5] *N* = 103 versus 81	Magrina et al. (2011) [6] *N* = 67 versus 37	Seamon et al. (2009) [12] *N* = 105 versus 76	Bell et al. (2008) [13] *N* = 40 versus 30
BMI (Kg/m^2^)	33 versus 29	30.7 versus 27.3*	34 versus 29*	33 versus 32
OP time (min)	191 versus 213*	182 versus 189	242 versus 287*	184 versus 171
EBL (mL)	75 versus 146*	141 versus 300*	100 versus 250*	166 versus 253*
LOS (day)	1.0 versus 1.2	1.9 versus 3.4*	1 versus 2*	2.0 versus 2.3
Nodes (*n*)	33 versus 23*	25 versus 27	21 versus 22	17 versus 17
Conversion (%)	2.9 versus 4.9	2.9 versus 10.8*	12 versus 26*	NA
Complication (%)	5.8 versus 13.6*	12 versus 14	13 versus 14	7.5 versus 20*

*P < .001 (mean values).
